# SWEEP: A Tool for Filtering High-Quality SNPs in Polyploid Crops

**DOI:** 10.1534/g3.115.019703

**Published:** 2015-07-06

**Authors:** Josh P. Clevenger, Peggy Ozias-Akins

**Affiliations:** Institute of Plant Breeding, Genetics and Genomics, The University of Georgia, Tifton, Georgia 31793

**Keywords:** SNP, polyploidy, peanut

## Abstract

High-throughput next-generation sequence-based genotyping and single nucleotide polymorphism (SNP) detection opens the door for emerging genomics-based breeding strategies such as genome-wide association analysis and genomic selection. In polyploids, SNP detection is confounded by a highly similar homeologous sequence where a polymorphism between subgenomes must be differentiated from a SNP. We have developed and implemented a novel tool called SWEEP: Sliding Window Extraction of Explicit Polymorphisms. SWEEP uses subgenome polymorphism haplotypes as contrast to identify true SNPs between genotypes. The tool is a single command script that calls a series of modules based on user-defined options and takes sorted/indexed bam files or vcf files as input. Filtering options are highly flexible and include filtering based on sequence depth, alternate allele ratio, and SNP quality on top of the SWEEP filtering procedure. Using real and simulated data we show that SWEEP outperforms current SNP filtering methods for polyploids. SWEEP can be used for high-quality SNP discovery in polyploid crops.

Single nucleotide polymorphism (SNP) markers are powerful genomic tools because they are ubiquitous in the genome, codominant, and can be in coding regions and act as functional markers. In diploid organisms, including plants, SNPs have become the preferred marker type in genome-wide studies (Rafalski 2002; Shirasawa *et al.* 2013; Wallace *et al.* 2014; Lee *et al.* 2015). In polyploid crops, however, accurate SNP detection becomes challenging due to the ambiguous mapping of highly similar homeologous loci, which increases the false-positive rate of detected SNPs. Despite this difficulty, SNPs have been utilized in some auto-polyploid and allo-polyploid crops with great success (Li *et al.* 2012; Byers *et al.* 2012; Ersoz *et al.* 2012; Cavanagh *et al.* 2013; Nagy *et al.* 2013; Uitdewilligen *et al.* 2013; Huang *et al.* 2013; Clevenger *et al.* 2015). In peanut, SNP discovery and utilization as markers have been difficult. There is promise in using SNPs from wild diploid progenitor species to genotype cultivated germplasm when breeding with populations incorporating wild introgressions (Bertioli *et al.* 2014), but SNP detection within cultivated germplasm has been limited. There has been one SNP-based *A. hypogaea* genetic map (Zhou *et al.* 2014) and one genetic diversity study using SNP markers (Khera *et al.* 2013) published. Compared to other species, the number of SNP markers in those two studies was low, with 1621 and 96 SNPs, respectively. In contrast, from a 180K SNP array in soybean, as many as 50,000 SNPs are polymorphic in a biparental population (Lee *et al.* 2015). In potato, an 8300 SNP array was developed from a set of 69,011 high-quality SNPs identified from six cultivars (Hamilton *et al.* 2011). Discovery of SNPs, array design, and GBS application has been successful in allohexaploid wheat (Cavanagh *et al.* 2013, Poland *et al.* 2012). This success has been due to using highly stringent mapping parameters for subgenome-specific mapping. Essentially, if subgenomes are diverged by 3%, only allowing reads that map with less than 3% differences will result in reads mapping to the correct subgenome and will alleviate the problem with false positives. In peanut, this strategy is not useful because the subgenomes comprising cultivated peanut are too similar.

Although the exact age of the hybridization event between probable progenitor species *Arachis duranensis* (A genome) and *Arachis ipaensis* (B genome) that led to the origin of cultivated peanut is not known, these two diploids diverged from one another only 3–3.5 million years ago (Nielen *et al.* 2012; Bertioli *et al.* 2013). As a comparison, the A and D genomes of cultivated cotton diverged between 5 and 10 million years ago (Wendel 1989), and the A, B, and D genomes of allohexaploid wheat diverged approximately 6.9 million years ago (Devos *et al.* 2005). Their recent divergence exacerbates the problem of detecting false-positive SNP calls that are manifested by polymorphism between subgenomes. Khera *et al.* (2013) could only utilize 96 out of 1536 selected SNPs after filtering for a false-positive rate of 93.8%. The false-positive SNP calls were actually polymorphisms between subgenomes and not between accessions. This was shown nicely as Leal-Bertioli *et al.* (2015) used these false-positive SNP calls to find evidence for tetrasomic recombination in *Arachis hypogaea*. Zhou *et al.* (2014) were only able to map 1621 out of 14,663 selected SNPs after filtering for a false-positive rate of 89%. It is clear that for SNP identification in peanut, new strategies need to be developed. Historical filtering strategies that have been successfully applied to other polyploid crops still result in excessive false-positive SNP calls in cultivated peanut.

Here, we describe a novel SNP filtering method called SWEEP (Sliding Window Extraction of Explicit Polymorphisms) that can be used as a tool to filter out false positives from a set of SNP calls. Our method uses the ubiquitous false-positive SNP calls and transforms them from a weakness to a strength by using their information to pull out the true SNPs that are polymorphic between genotypes of interest. SWEEP is implemented in a Perl script that is easy to use; the user only needs to supply sorted and indexed bam files and the reference genome used to map sequence reads. In addition, previously generated vcf files can be used. SWEEP will call SNPs using Samtools (Li *et al.* 2009) and implement our novel filtering method. If desired, then the user can filter further based on additional common metrics. We show the accuracy and efficacy of our method in cultivated peanut by calling SNPs in leaf transcriptome RNAseq data from six *Arachis hypogaea* genotypes and also on simulated data. SWEEP is also applicable for other allopolyploid crops.

## Materials and Methods

### Leaf tissue RNA-seq and read mapping

Six accessions that are parents of F6:8 recombinant inbred line populations were assayed: Tifrunner (TR); Florida-07 (F07); SPT06-06 (SPT); NC 3033 (NC); New Mexico Valencia A (NM); and C76-16 (C76). SPT06-06 has been released as GP-NC WS 16 (Tallury *et al.* 2014). These data were previously published in Clevenger *et al.* (2015).

Raw reads were trimmed for adaptor sequences. Sequence quality was assessed using FASTQC (http://www.bioinformatics.babraham.ac.uk/projects/fastqc/) and reads were further trimmed for base quality (>30) and base usage bias. Due to nonrandom base usage in the first 10 bases of these libraries, 10 bases were trimmed from the 5′ end. Trimmed libraries were then filtered for rRNA contamination by mapping to a set of known rRNA sequences using Bowtie (Langmead *et al.* 2009) and allowing two mismatches in the 25 bp seed.

A *de novo* assembly was constructed using New Mexico Valencia A and the Trinity software package for *de novo* assembly (Haas *et al.* 2013) with the following parameters: "–normalize_reads–CuffFly–min_glue 4." The assembly was then filtered for redundancy if any transcript covered more than 75% of a longer transcript with more than 95% nucleotide identity. Final assembly statistics are in Supporting Information, Table S1.

Processed sequences for each genotype were first normalized to a maximum kmer coverage of 30 using the insilico_read_normalization.pl script of Trinity. Normalized reads were then mapped to the *de novo* assembly using Bowtie2 (Langmead and Salzberg 2012) and the following parameters to only allow proper and complete read pairs mapping with no clipping: "–end-to-end–no-mixed." Average read mapping across all libraries was 59.62% with 16.95% unique mapping. Mapping statistics are shown in Table S2. SNPs were called using Samtools mpileup with default parameters (Li *et al.* 2009). No filtering is used in this step because all possible SNPs will subsequently be filtered using our SWEEP algorithm. SWEEP was used to filter called SNPs using default parameters. SWEEP is available on Github at https://github.com/jclev-uga/SWEEP/ as free software.

### Traditional filtering

For the purposes of comparison with SWEEP, we have compiled a consensus filtering procedure. These criteria include filtering for mean read depth across all samples covering a SNP to be at least four reads, for minor allele frequency greater than 0.35, for SNP quality >30, and for SNPs that are not within 35 bp of another SNP. To accomplish these filtering criteria we have used the freely available software, vcftools (Danecek *et al.* 2011), and custom scripts.

VCF files were first filtered for depth (four reads per genotype covering base of interest) using a custom python script. Then vcftools (Danecek *et al.* 2011) was run with the following parameters: "–maf 0.35–minQ 30–remove-filtered-all–remove-indels –recode." Finally, SNPs within 35 bp of another called SNP were filtered out using a custom python script.

### SNP validation by SANGER sequencing

A set of 28 randomly chosen SNPs were selected for validation using Sanger sequencing. Sequence was extracted from 300 bases upstream and downstream of the SNP of interest and primers were designed with the SNP in the center of the product using primer3 (Untergrasser *et al.* 2012). Two accessions called as reference and two accessions called as alternate were sequenced for validation.

### Analysis of SWEEP-filtered SNPs

All analyses of polymorphic SNPs and false-positive rates were performed using custom python scripts. False-positive rate was measured by using the Samtools-derived genotype probabilities for calling genotypes and then searching for the following genotypic pattern among the sets of five, four, three, and two genotypes:

**If** every genotype has reads with the alternate base at the site of interest, then the called SNP is a false positive.
**If** at least one genotype has all reads with the reference base at the site of interest, then the called SNP is determined to be a true allelic SNP.

### Simulation

*De novo* assembled transcripts of New Mexico Valencia A were reduced to a nonredundant set of 28,967 transcripts using the Evigene pipeline (http://arthropods.eugenes.org/genes2/about/EvidentialGene_trassembly_pipe.html) (Nakasugi *et al.* 2014). A copied set of transcripts was then mutated randomly at 1% divergence using a custom python script. Illumina reads were simulated at 20× coverage of the mutated set of transcripts using ART (Huang *et al.* 2012) and mapped back to the original set of transcripts to identify the induced "homeologous" mutations. The set of original transcripts and the mutated set were then used to generate three genotypes, mutated randomly again at 0.01%, 0.02%, and 0.0001% divergence. Reads were simulated using ART 100 bp paired-end reads with an insertion size of 250 bp at 5×, 10×, 15×, and 20× coverage. Reads for each simulated genotype for all coverages were mapped to the original set of 28,967 transcripts and SNPs were called using Samtools mpileup with default parameters. GATK UnifiedGenotyper was used with the following parameters: "-stand_call_conf 30 –ploidy 4." Traditional filtering methods were performed as above. SWEEP filtering was performed with the following parameters: "-s 1 -d 5 -r 0 –ultimate."

### Figure construction

All graphs were made using R statistical software (r-project.org) and the package ggplot2.

### Data availability

SWEEP is freely available under the MIT license at https://github.com/jclev-uga/SWEEP/. Any other scripts used in this study for filtering, simulation, etc. are available upon request. Supplementary Information contains Table S1, Table S2, and Table S3.

## Results and Discussion

### Sweep

SWEEP is a Perl wrapper script that calls a series of python scripts to implement the sliding window strategy of identifying true allelic SNPs between polyploid genotypes. We define "allelic" SNP in a polyploid crop as originating from the same subgenome, within the same locus, and being polymorphic between genotypes. In contrast, a homeologous SNP we define as a polymorphism that exists at homeologous loci and is polymorphic between subgenomes within genotypes. The user can input sorted and indexed bam files from as many genotypes as needed, and SWEEP will use Samtools to call the SNPs, filter them using the sliding window strategy, and filter them based on metrics that can be good identifiers of high-quality SNP calls (Clevenger *et al.* 2015). Additionally, the user can input a vcf file generated by their tool of choice and SWEEP will filter the SNPs based on the supplied options. These metrics include genotypic likelihood, read depth, and ratio of reference base to alternate base containing reads spanning the locus.

### Sliding window procedure

**Figure 1:**
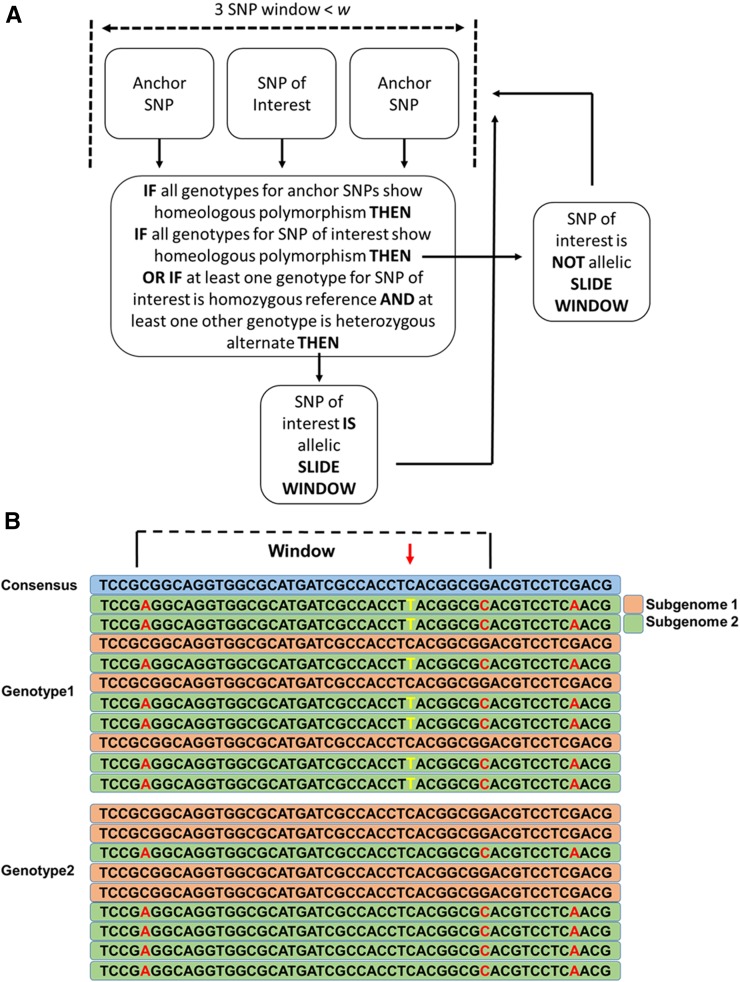
(A) Logic for SWEEP pipeline. (B) Example of detection of a SNP between genotypes. Blue bar represents the reference consensus sequence. Green bars represent one subgenome-derived sequence. Orange bars represent the alternative subgenome-derived sequence. Bases in red are within genome polymorphisms and in this instance are the anchor SNPs. Bases in yellow are the true between-genotype SNPs.

SWEEP tackles the uniquely polyploid issue of detecting false-positive SNPs that are polymorphisms between subgenomes. To do this, the program visits each called SNP using a sliding window of three called SNPs within a set window size. The user can adjust the size of this window. It is recommended that the size of this window should be set to the average length of the sequenced reads. Confidence in the actual sequence of that locus that the read represents declines if not contained in a continuous read. Ends of reads have a higher percentage of sequencing errors, so further confidence can be attained by setting a smaller window size (Song *et al.* 2014). The SNP of interest is anchored by called SNPs up-stream and down-stream. The procedure checks all genotypes and looks for a case where: (1) the SNP of interest is homozygous for the reference allele in at least one genotype while (2) the "anchor" SNPs are always heterozygous alternate allele. If a SNP of interest lies at the end of a known sequence or only has one anchor SNP within the window size, then SWEEP will use only one anchor. The elegance of the procedure is that it uses the false-positive, homeologous SNPs as a guide to find the true SNPs. A flow chart of the procedure is shown in Figure 1A and a representative sequence example with a detected allelic SNP is shown in Figure 1B.

### User-determined filtering

Samtools mpileup calculates the phred scale genotype probabilities for each SNP in the following format: AA/AB/BB, where A represents the reference allele and B represents the alternative allele. A value of 0 is the highest probability that the genotype represented is correct and the least likely probability will have a value of 255. For example, 0,255,255 represents the highest probability that the sample is homozygous for the reference allele. These scores are calculated based on a Bayesian statistical model for characteristics of the mapped reads that cover the base in question, including mapping quality, base quality, etc. The SWEEP filtering option is simple, allowing three levels of stringency for filtering. The default filters out homozygous genotype calls if the phred-based score of a heterozygous call is less than 20, medium stringency filters at less than 125, and high stringency filters at less than 200. The lowest stringency filter was used on the set of SNPs selected for sequencing and produces robust results. Higher stringency can be used when the number of SNPs between genotypes is high and only the best SNPs are needed.

Read depth can be an important filter for high-quality SNP calls. It is more critical in an allopolyploid context where sufficient sequence information is needed to discern homeologous read mapping. SWEEP allows the user to filter based on per sample average read depth. For example, setting the option to "4" will filter out any SNPs that have less than four reads per sample covering the base.

Increasing the ratio increases the likelihood that the alternate allele is present in more than one genotype, which will reduce rare SNPs. In the context of SNP detection in polyploids from next-generation sequence data, confidence in the veracity of a rare SNP is low. This filter is best utilized when the number of genotypes assayed is high and total SNPs is not a concern.

### The –ultimate filter

SWEEP gives the user the option to include the ultimate filter. This option is more computationally intensive and will require longer run times (Table S3). The default SWEEP program relies heavily on Samtools-calculated genotype probabilities. Manual inspection of alignments revealed that in some cases, reads map to the locus with the alternate base even when the genotype probability score of 0 for homozygous reference is calculated. These reads may have lower mapping quality or have been clipped. In the case of a SNP where only one genotype has been called homozygous reference, it is important to make sure no single read in the experiment from that genotype contains the alternate base, no matter the mapping quality of the read. These cases can cause false-positive SNP calls and lead to spurious downstream analysis. As an alternative to manually checking every individual SNP, the user can implement the –ultimate function, which, after SWEEP filtering, checks every base from a genotype covering the SNP of interest that was called homozygous reference. If one read covering the SNP contains the alternate allele, then the SNP is filtered out. Running –ultimate is recommended in all cases for optimal quality.

### Leaf transcriptome case study

As a case study we used RNAseq data from leaf tissue of six *A. hypogaea* genotypes (Clevenger *et al.* 2015). RNAseq data are suited for SNP discovery in repetitive, complex genomes due to inherent complexity reduction and have been used successfully in many polyploid crops (Clevenger *et al.* 2015). As a reference we used a *de novo* assembled transcriptome of New Mexico Valencia A, the genotype with the highest number of sequenced reads.

### SWEEP filtering

Samtools called a total of 610,942 SNPs relative to the *de novo* reference transcriptome. After SWEEP filtering with default parameters, a total of 49,876 SNPs was retained. After using the –ultimate function, a final set of 5025 SNPs was retained. This result shows the importance of the –ultimate function because in some cases it is difficult to select a confident genotype likelihood cut-off. We selected 50 random SNP loci to validate for Sanger sequencing. We sequenced two genotypes that were designated as reference and two genotypes that were designated as alternate. Out of the 50 sequenced loci, we obtained reliable sequence data for 28. Of the 28 loci, 25 were confirmed as true SNPs called using SWEEP for all genotypes and three were false positives for 89% accuracy. During pilot experiments, we sequenced 21 randomly chosen loci and confirmed 17 of them for 85% accuracy (Clevenger *et al.* 2014). Combined, these data represent 49 sequenced loci from four different genotypes per locus with a combined 86% accuracy.

### Traditional filtering

Traditionally, SNP identification in polyploid crops has relied on filtering using a set of criteria that has been identified as representing how a true SNP will be described in a SNP call (Clevenger *et al.* 2015). For purposes of comparison with SWEEP, we have compiled a consensus filtering procedure relying on read depth, SNP quality, minor allele frequency, and physical proximity to adjacent called SNPs. Traditional filtering resulted in 69,874 of the 610,942 called SNPs being retained.

### SWEEP *vs.* traditional filtering

**Figure 2:**
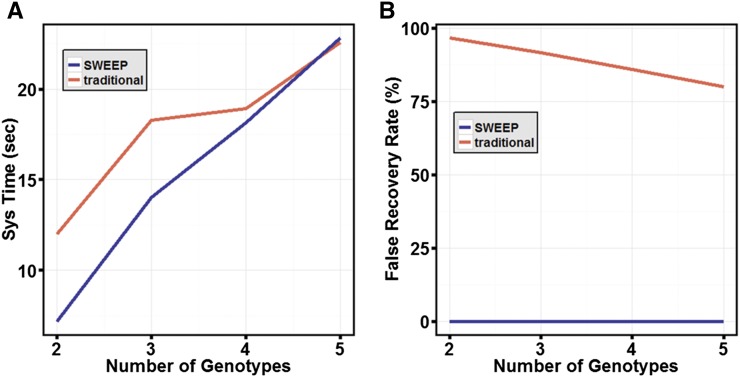
SWEEP filtering *vs.* traditional filtering methods. (A) Samtools-called SNPs were filtered using vcftools and SWEEP and evaluated for computational time using combinations of all five, four, three, and two genotypes. (B) SWEEP filtering and traditional filtering were evaluated for false-positive rate using combinations of all five, four, three, and two genotypes.

**Figure 3:**
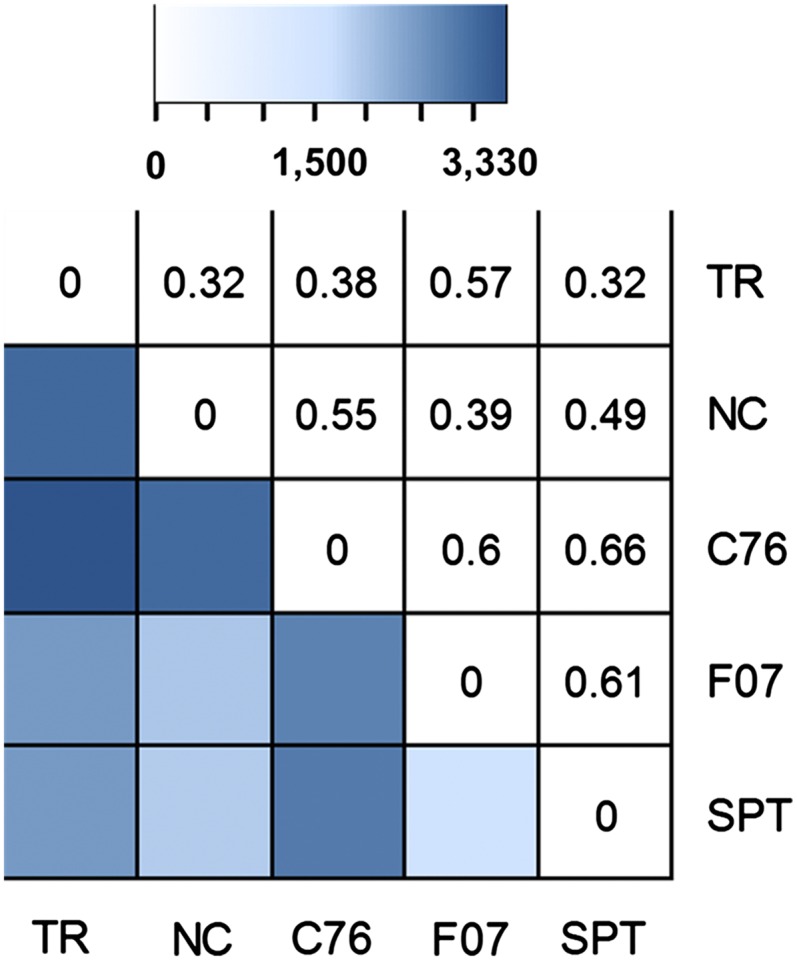
Pairwise polymorphism between genotypes. The upper diagonal above shows pairwise fraction of polymorphic SNPs relative to total SNPs called. The heatmap in the lower diagonal reflects the range of pairwise polymorphic SNPs. Genotypes are Tifrunner (TR), NC3033 (NC), C76-16 (C76), Florida-07 (F07), and SPT06-06 (SPT).

A comparison between the computational times for vcftools filtering and SWEEP of different numbers of genotypes showed that the two methods are comparable (Figure 2A). The –ultimate function requires more computational time and is an optional quality check (Table S3). It is important to test for the efficacy of the filtering method. Without a known set of validated SNPs, we cannot fully verify the SNPs identified *in silico*, but we can evaluate them based on a set of assumptions. The assumption we make is that given a locus with a minimum number of reads mapping to it, if all the reads mapping to that locus have the reference base for a particular genotype, then that genotype does not have a SNP at that position. If, however, every genotype assayed contains at least one read that exhibits the alternate base at the position of interest, then we can reliably say that the base of interest is showing either a polymorphism in all genotypes relative to the reference or a polymorphism between subgenomes. In the latter case the SNP is not a true allelic SNP and can be determined to be a false positive. We compared the SWEEP filtering method with the traditional filtering method based on these assumptions and using different numbers of genotypes (Figure 2B). For SWEEP, the false-positive rate is near zero, ranging from 0.01% for five genotypes and 0.02% for two genotypes (Figure 2B). Using the traditional filtering method, the false positive rate is, in contrast, very high, ranging from 80% for five genotypes and 96.9% for two genotypes. SWEEP filtering results in good polymorphism between genotypes. Figure 3 shows the polymorphism among detected SNPs with SWEEP. The pairwise SNP polymorphisms between genotypes ranged from 32% to 66%.

### SWEEP *vs.* traditional filtering simulation

**Figure 4:**
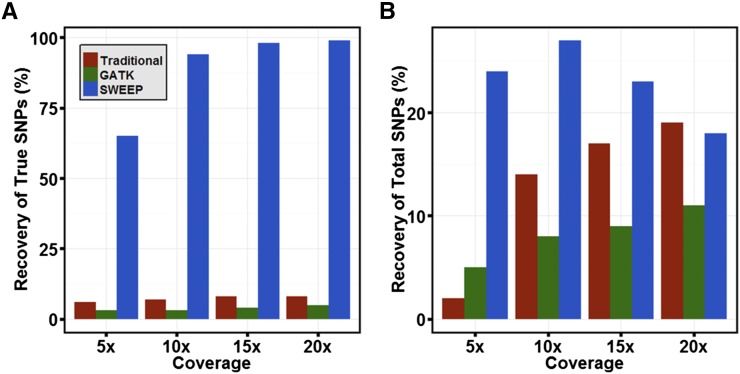
Percentage of true SNPs compared to false-positive, homeologous SNPs called by filtering method. Samtools and GATK with traditional filtering methods were compared to SWEEP filtering in a simulation with 5×, 10×, 15×, and 20× coverage. (A) Recovery of true SNPs as percentage of total SNPs retained after SWEEP filtering and traditional filtering using GATK and Samtools. (B) Recovery of true SNPs as percentage of total simulated true SNPs.

To show that SWEEP filtering performs better in an allopolyploid context using a set of known SNPs, we performed a simulation. For the simulation we used a compressed set of 28,967 transcripts after redundancy reduction using the Evigene pipeline (http://arthropods.eugenes.org/genes2/about/EvidentialGene_trassembly_pipe.html) (Nakasugi *et al.* 2014). We randomly mutated these transcripts with a divergence of 1% to form a set of homeologous transcripts; therefore, the location of all homeologous polymorphisms was predetermined. From these two sets we generated three distinct genotypes: one genotype contained additional SNPs at 0.01% divergence; a second genotype contained unique SNPs at 0.02% divergence; and the third genotype maintained the SNPs from genotype two and contained a unique set at 0.0001% divergence. Simulated Illumina reads from these three genotypes were mapped to the original set of 28,967 nonmutated transcripts with 5×, 10×, 15×, and 20× coverage. This scenario mimics a situation in which the reference a researcher is using contains “collapsed” sequences representing a consensus sequence between homeologous or paralogous genes. We evaluated SWEEP filtering against Samtools with traditional filtering and GATK (ploidy set to 4×) with traditional filtering (Figure 4A). SWEEP filtering greatly outperformed both methods for percentage of SNPs recovered that are true SNPs relative to homeologous SNPs. At 5× coverage, SWEEP recovers true SNPs at a rate of 65%, and that increases to 94% at 10× coverage, 98% at 15× coverage, and 99% at 20× coverage. Samtools with traditional filtering surprisingly outperformed GATK with the ploidy option set to 4× with 8% true SNP recovery at 20× coverage. Neither Samtools nor GATK with traditional filtering recovered true SNPs at higher than 8% with any coverage. The lower percentage of true SNPs identified by SWEEP at 5× coverage results from lack of sampling both homeologous loci at low sequence coverage. In such cases, there is no way to discern, within the experiment, a homeologous SNP from an allelic SNP. The higher percentage of true SNPs recovered for SWEEP is not a consequence of identifying fewer true SNPs overall because SWEEP recovered a larger number of true SNPs than Samtools and GATK at 5×, 10×, and 15× coverage and retained a comparable amount to Samtools at 20× coverage (Figure 4B).

### Summary and Conclusions

We have shown that SWEEP is a reliable method for identifying true allelic SNPs among genotypes of *A. hypogaea* and is a major improvement of current methods. We have validated SWEEP-filtered SNPs at an accuracy rate of 85%. SWEEP outperforms traditional filtering methods relative to the false-positive rate. The false-positive rate of traditionally filtered SNPs ranges from 80% to 96%, whereas the false-positive rate of SWEEP-filtered SNPs ranges from 0.01% to 0.02%. Using a simulation, we further validated that SWEEP greatly outperforms current filtering methods, retaining a high percentage of true SNPs relative to homeologous polymorphisms (65%–99% depending on coverage) compared to other methods (2%–8%). A similar method was developed and implemented in parallel to design high-quality probes for a 90k SNP array for allo-octoploid cultivated strawberry *Fragaria* × *ananassa* (Bassil *et al.* 2015). Their method uses "destabilization" sites as a method of ploidy reduction in their array design. These destabilization sites are similar to our anchor SNPs in our sliding window design and confirm that using homeologous polymorphism haplotypes as contrast is an efficacious method for discerning true genotypic SNPs in polyploid species. Using SWEEP for outcrossing polyploids or autopolyploids with higher levels of ploidy may not be useful. However, it is clear from these data that SNP identification in peanut cannot rely on traditional methods and that SWEEP is a reliable tool for genotyping using GBS and for identifying SNPs for SNP array design for polyploids.

## 

## Supplementary Material

Supporting Information
