# Supplementary material for: SWEEP: A Tool for Filtering High-Quality SNPs in Polyploid Crops
Source: G3 (Bethesda). 2015 Jul 6;5(9):1797–803. doi: 10.1534/g3.115.019703 (PMC4555216; doi:10.1534/g3.115.019703)
Supplement: Supporting Information [file supp_5_9_1797__index.html]

SWEEP: A Tool for Filtering High-Quality SNPs in Polyploid Crops — Supporting Information 

# SWEEP: A Tool for Filtering High-Quality SNPs in Polyploid Crops

## Supporting Information for Clevenger and Ozias-Akins, 2015

**Files in this Data Supplement:**

- Supporting Information - Tables S1-S3 (PDF, 172 KB)
- Table S1 - Assembly statistics of *de novo* Trinity assembly. (PDF, 109 KB)
- Table S2 - Sequence statistics for all genotypes sequenced in this study. (PDF, 108 KB)
- Table S3 - Sys time for -ultimate filtering using one 8-core node (Intel Xeon processors) and 32 Gb of RAM. (PDF, 81 KB)
