# Supplementary material for: SWEEP: A Tool for Filtering High-Quality SNPs in Polyploid Crops
Source: G3 (Bethesda). 2015 Jul 6;5(9):1797–803. doi: 10.1534/g3.115.019703 (PMC4555216; doi:10.1534/g3.115.019703)
Supplement: Supporting Information [file supp_g3.115.019703_019703SI.pdf]

G3 · Genes | Genomes | Genetics

SWEEP: a tool for filtering high quality SNPs in polyploid crops.

Josh P. Clevenger[1] and Peggy Ozias-Akins[1*]

[1]Institute of Plant Breeding, Genetics & Genomics, The University of Georgia, Tifton, GA 31793.

*Corresponding author: The University of Georgia, Department of Horticulture, 2356 Rainwater Road, Tifton, GA 31794. Email: pozias@uga.edu

DOI: 10.1534/g3.115.019703

Table S1 Assembly statistics of *de novo* Trinity assembly

	**New Mexico Valencia A Assembly**
Transcripts	124,357
N50 (bp)	1,927
Average (bp)	1,118
Total Assembled (bp)	139,090,891

Table S2 Sequence statistics for all genotypes sequenced in this study. Percentage of mapped reads are to the New Mexico Valencia A *de novo* assembly

**Genotype**	**Total Reads**	**Mapped Reads**
		**%**
Florida 07	13,881,744	60.30
NC3033	15,736,991	55.98
SPT06-06	16,252,634	56.48
Tifrunner	35,457,450	61.97
C76-16	9,949,147	63.36

Table S3 Sys time for –ultimate filtering using one 8-core node (Intel Xeon processors) and 32 Gb of RAM.

Genotypes	sys (sec)	sys (min)	Total SNPs visited	Rate (SNP/sec)
5	18,121	302.02	49,876	2.75
4	15,020	250.33	47,227	3.14
3	10,207	170.12	26,052	2.55
2	7,542	125.70	16,880	2.24
