# Supplementary material for: SWEEP: A Tool for Filtering High-Quality SNPs in Polyploid Crops
Source: G3 (Bethesda). 2015 Jul 6;5(9):1797–803. doi: 10.1534/g3.115.019703 (PMC4555216; doi:10.1534/g3.115.019703)
Supplement: Supporting Information [file supp_g3.115.019703_TableS1.pdf]

Table S1 Assembly statistics of *de novo* Trinity assembly

	**New Mexico Valencia A Assembly**
Transcripts	124,357
N50 (bp)	1,927
Average (bp)	1,118
Total Assembled (bp)	139,090,891
