# Supplementary material for: SWEEP: A Tool for Filtering High-Quality SNPs in Polyploid Crops
Source: G3 (Bethesda). 2015 Jul 6;5(9):1797–803. doi: 10.1534/g3.115.019703 (PMC4555216; doi:10.1534/g3.115.019703)
Supplement: Supporting Information [file supp_g3.115.019703_TableS2.pdf]

Table S2 Sequence statistics for all genotypes sequenced in this study. Percentage of mapped reads are to the New Mexico Valencia A *de novo* assembly

Genotype	Total Reads	Mapped Reads
		%
Florida 07	13,881,744	60.30
NC3033	15,736,991	55.98
SPT06-06	16,252,634	56.48
Tifrunner	35,457,450	61.97
C76-16	9,949,147	63.36
