# Supplementary material for: SWEEP: A Tool for Filtering High-Quality SNPs in Polyploid Crops
Source: G3 (Bethesda). 2015 Jul 6;5(9):1797–803. doi: 10.1534/g3.115.019703 (PMC4555216; doi:10.1534/g3.115.019703)
Supplement: Supporting Information [file supp_g3.115.019703_TableS3.pdf]

Table S3 Sys time for –ultimate filtering using one 8-core node (Intel Xeon processors) and 32 Gb of RAM.

Genotypes	sys (sec)	sys (min)	Total SNPs visited	Rate (SNP/sec)
5	18,121	302.02	49,876	2.75
4	15,020	250.33	47,227	3.14
3	10,207	170.12	26,052	2.55
2	7,542	125.70	16,880	2.24
